# Biomechanical Comparison of Asymmetric Implant Configurations for All-on-Four Treatment Using Three-Dimensional Finite Element Analysis

**DOI:** 10.3390/life12121963

**Published:** 2022-11-23

**Authors:** Onur Gönül, Ahmet Çicek, İbrahim Murat Afat, Emine Tuna Akdoğan, Onur Atalı

**Affiliations:** 1 Department of Oral and Maxillofacial Surgery, Faculty of Dentistry, Marmara University, 34722 İstanbul, Turkey; 2 Department of Oral and Maxillofacial Surgery, Faculty of Dentistry, İstanbul Kent University, 34433 İstanbul, Turkey

**Keywords:** dental implants, finite element analysis, prosthodontics

## Abstract

**Simple Summary:**

The all-on-four concept, in which two implants are placed vertically in the anterior region and two at an angle in the posterior region, reduces the need for multi-stage surgical procedures while successfully rehabilitating complete edentulism. The aim of this study is to examine the effect of unilaterally more posterior placement of implants applied according to the all-on-four concept on the stress distribution on bone, implants and other prosthetic components, using the finite element analysis method. Findings of this study suggest that placing the implant further posterior to first molar region may prevent the bone resorption that occurs with high stress around the crestal bone. However, increased stress on the implants and prosthetic parts may lead to failures.

**Abstract:**

The aim of this study is to examine the effect of unilaterally more posterior placement of implants (Straumann BLT 4.1 mm in diameter and 12 mm long) applied according to the all-on-four concept on the stress distribution on bone, implants, and other prosthetic components, using the finite element analysis method. Three scenarios were modelled: For Model 1 (M1), anterior implants were placed symmetrically perpendicular to the bone in the right and left lateral incisor region, while the necks of the posterior implants placed symmetrically in the second premolar region were angled at 30 degrees. For Model 2 (M2) the implant in the left second premolar region was placed to the first molar region unilaterally. For Model 3 (M3) the implant in the left lateral incisor region was placed to the canine region unilaterally. Vertical and oblique forces (100 N) were applied in the right first molar region. The von Mises and maximum (Pmax) and minimum (Pmin) principal stresses were obtained. The highest stress concentration on the cortical bone was observed in the second premolar region in all models when oblique forces were applied. M1 was highest (8.992 MPa) followed closely by M3 (8.780 MPa) and M2 was lowest (3.692 MPa). The highest stress concentration on the prosthetic parts was observed in this framework when oblique forces were applied. M2 was highest (621.43 MPa) followed by M3 (409.16 MPa) and the lowest was M1 (309.43 MPa). It is thought that placing the implant further posterior to first molar region may prevent the bone resorption that occurs with high stress around the crestal bone. However, increased stress on the implants and prosthetic parts may lead to failures.

## 1. Introduction

Complete edentulism is one of the most complex dental problems. With the development of treatment options and increasing patient expectations, solutions based on dental implants are generally adopted [1]. However, the anatomy is one of the most important factors limiting dental implant applications in edentulous patients. In the mandible, the inferior alveolar nerve limits implant application, while the limiting structure in the maxilla is usually the maxillary sinuses. These issues can be resolved by various procedures, such as sinus lifting, onlay grafting, or inferior alveolar nerve repositioning. However, procedures also have limitations. Many procedures prolong the treatment time and may require repeated surgical intervention depending on local factors. The treatment cost may increase and complications may develop. All of these factors reduce the acceptability of the procedures for patients and lead to the use of less invasive procedures [2].

In 1990, Maló et al. introduced the all-on-four concept, in which two implants are placed vertically in the anterior region and two at an angle in the posterior region, thus reducing the need for multi-stage surgical procedures while successfully rehabilitating patients with low bone volume. The use of the all-on-four concept has increased in years because it is favorable in terms of patient morbidity, treatment time, and cost [2,3].

In the all-on-four concept, anterior implants are generally placed in the lateral incisor region, while the posterior implants are angled so that the implant neck emerges from the second premolar region [2,4,5]. This keeps both the distance between implants and the cantilever distance short. However, this standard treatment plan cannot be applied in patients with highly resorbed crest areas or asymmetric sinus pneumatisation. Providing the right biomechanical conditions is very important for this treatment and requires accurate adjustment of the prosthetic conditions, implant positions, and occlusion. In this respect, finite element analysis has been a widely accepted tool that can be used to gain insight into the biomechanical behavior of the analyzed structures, allowing for estimation of implant position, dimensions, and angulations to determine safe parameters for their clinical use in all-on- four treatments. In the literature, many studies used finite element analysis to examine the effect of changing implant positions, dimensions, and angulations on bone and prosthetic parts [2,4,6,7,8,9,10,11,12].

Kumari et.al evaluate stress distribution on implants in all-on-four placements with varying distal implant angulations (30-, 40-, 45-degree) and in maxilla using finite element analysis. The 45-degree tilt induced higher stress values at the bone–implant interface, especially in the distal aspect, than the other two tilts analyzed [5].

Begg et al. reported that the peri-implant bone surrounding the 45-degree-angled distal abutment may be more prone to occlusal overload than the bone surrounding the implants with lesser tilts (17- and 30-degree) [13].

Bhering et al. reported three framework materials were evaluated: cobalt-chrome (CoCr), titanium (Ti), and zirconia (Zr), totalizing six groups. A unilateral oblique force of 150 N was applied to the posterior teeth. Stiffer materials (CoCr and Zr) have the most favorable biomechanical behavior and decrease the stress levels for bone, implants, screws, abutments, and displacement magnitude. The use of titanium as a prosthetic framework material in the all-on-four treatment concept exhibited the worst biomechanical behavior. Ti presented the highest stress values on the cortical bone, implants, abutments, prosthetic screws, and displacement levels [14].

All of these studies used symmetrically placed implant models. Increasing the distance between implants, or asymmetric placement of implants can cause higher stresses on bone, implants, and other prosthetic components. These high stress areas may lead to failures. To investigate this hypothesis, we performed stress analyses in three different all-on-four scenarios using finite element analysis.

## 2. Materials and Methods

**Figure 1:**
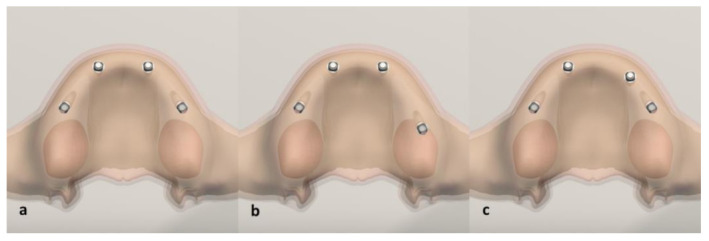
Occlusal view of digital model scenarios with different implant placements (**a**) M1, (**b**) M2 and (**c**) M3.

In this study, two implants were placed in the anterior region and two in the posterior region, in accordance with the all-on-four treatment concept in the edentulous maxilla in a virtual environment. The location of one implant on the alveolar crest was varied among the scenarios and the stress changes were measured (Figure 1).

**Figure 2:**
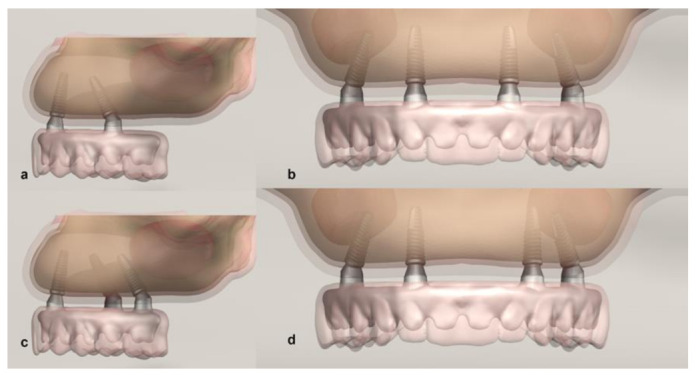
Digital model scenarios with different implant placements (**a**) Lateral M1, (**b**) Frontal M1, (**c**) Lateral M2 and (**d**) Frontal M3.

A hybrid prosthesis was attached to the applied implants with multiunit abutments (Figure 2).

**Figure 3:**
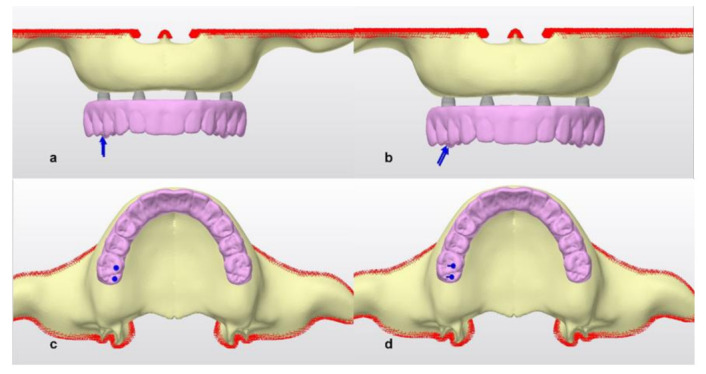
Vertical and oblique force vectors (**a**) frontal view vertical force (**b**) frontal view oblique force (**c**) occlusal view vertical force (**d**) occlusal view oblique force.

Then, vertical and oblique forces were applied to the prosthesis and the stresses on the framework, abutments, abutment screws, implants, and bone tissue around the implants were observed (Figure 3).

To create a geometric model of the upper jaw, tomography data of a fully edentulous adult patient were obtained from the Visible Human Project (US National Library of Medicine). The jawbone was scanned by cone beam tomography (ILUMA CBCT; 3M IMTEC, Ardmore, OK, USA) and the resulting sections were exported in DICOM 3.0 format and transferred to 3D-Doctor (Able Software, Lake Forest, CA, USA). Bone tissue images of these sections were separated by exporting the model in stl data format. The jaw model was adjusted with VRMesh software (VirtualGrid, Bellevue, WA, USA). An alveolar arch with a suitable volume for U-shaped implant placement was created. Cancellous bone was obtained from the bone tissue by the offset method. The periphery of the spongy bone was surrounded by 1-mm-thick cortical bone and 2-mm-thick mucous tissue was added to obtain a realistic image [7,14].

For editing, we homogenized the three-dimensional (3D) mesh, created a 3D solid model, and carried out a finite element stress analysis, and used an Activity 880 (smart optics Sensortechnik, Bochum, Germany) optical scanner, Rhinoceros 4.0 (Robert McNeel & Associates, Seattle, WA, USA) 3D modelling software, VRMesh Studio, and ALGOR FEMPRO (ALGOR, Pittsburgh, PA, USA).

After the models were created geometrically with VRMesh, they were exported into ALGOR FEMPRO in. stl format for the analysis. The elasticity modulus and Poisson ratio that define the physical properties of the structures are given in Table 1; these were used to introduce into the created model maxilla information compatible with the ALGOR software, and the material from which the prosthetic structures were made [5,10,15,16].

After scanning the implants to be used in the 3D maxillary models with the Activity 880 optical scanner, the implant [Straumann, bone level tapered (BLT)], abutment, and screw (Straumann Group, Basel, Switzerland) data were obtained and input into the maxilla model for the scenarios.

There were three different implant placements. Straumann BLT implants 4.1 mm in diameter and 12 mm long were used in all scenarios. For M1, anterior implants were placed symmetrically perpendicular to the bone in the right and left lateral incisor region, while the necks of the posterior implants placed symmetrically in the second premolar region were angled at 30 degrees. For M2, the anterior implants were the same as in M1, the right posterior implant neck was placed at 30 degrees, protruding from the second premolar region, but the left implant neck was placed in the first molar region at 30 degrees. In M3, anterior implants were placed in the right lateral incisor region and left canine tooth region, while both posterior implants were placed symmetrically, angled at 30 degrees in the second premolar region. Then, abutments were placed on the implants. Hybrid prostheses with metal substructures that ended at the first molars were placed on the abutments. After the prosthesis was placed, 100 N forces were applied vertically and obliquely (at 45 degrees) over the palatal tubercles of the right first molar [1,10,15].

The resulting stresses in the models were recorded as the maximum (Pmax) and minimum (Pmin) principle stresses for bone tissue. The stresses on ductile materials, such as prosthetic parts and implants, were recorded as Von Mises values and converted into visual data by color coding.

## 3. Results

**Figure 4:**
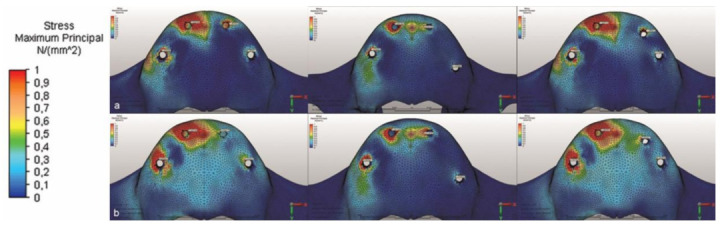
The P_max_ values, illustrated using a color scale under (**a**) vertical and (**b**) oblique forces.

The three different models were subject to 100 N vertical and oblique forces in the same region. The resulting stresses are shown using a color scale. After the forces were applied individually for each model, the high- and low-stress regions were evaluated. The highest Pmax on the cortical bone was around the implant in the right lateral incisor region in M1, followed closely by the right lateral incisor region in M3 (Table 2 and Figure 2). With an oblique load, the highest stress was in the implant neck in the second premolar region in M1, followed closely by the same implant in M3 (Table 2 and Figure 4). With vertical loading, Pmin was observed in all implant regions in M2. The situation was similar under oblique loading.

**Figure 5:**
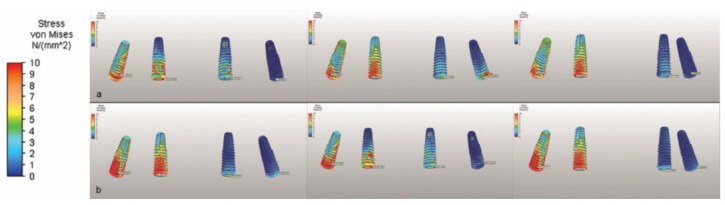
The Von Mises stress on implants, illustrated using a color scale under (**a**) vertical and (**b**) oblique forces.

Considering the stresses in the implant neck region, M2 had the highest Von Mises values under vertical loading in the right second premolar region. The highest value shifted to the implant in the right lateral incisor region under oblique loading (Table 3 and Figure 5).

**Figure 6:**
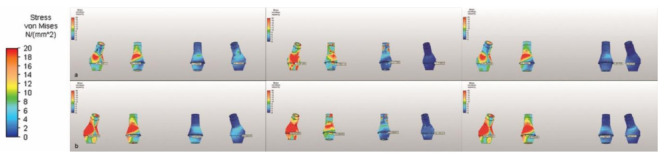
The Von Mises stresses on the abutment under (**a**) vertical and (**b**) oblique forces, illustrated using a color scale.

M2 had the highest Von Mises stresses under vertical loading in the abutment neck region. The highest value was seen in the implant in the second premolar region in M3. The abutment neck region had high Von Mises stresses under oblique loading, with the highest total stress occurring in M2, and the highest value in an implant area being that for the implant in the second premolar region of M1 (Table 4 and Figure 6).

**Figure 7:**
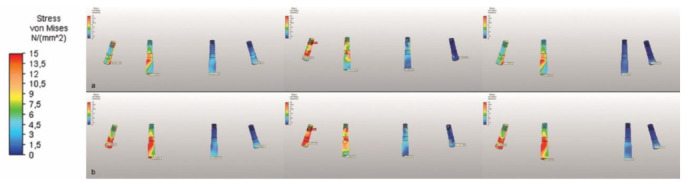
The Von Mises stresses on abutment screws under (**a**) vertical and (**b**) oblique forces, illustrated using a color scale.

The highest Von Mises stresses on the abutment screws under a vertical force were seen in M1, but the forces were evenly distributed on the screws. The highest value was in the second premolar region in M2. Overall, the highest total Von Mises stresses were observed in M1, and the highest value in an implant area was that for the screw in the second premolar region in M3, followed closely by the implant in the same region in M1 (Table 5 and Figure 7).

**Figure 8:**
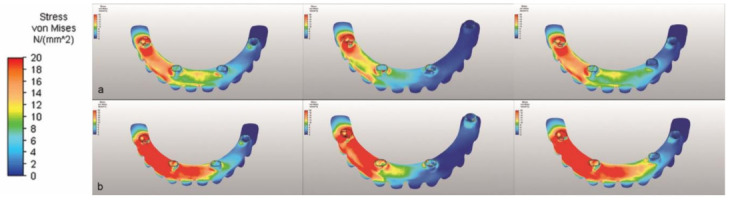
The Von Mises stresses on the framework under (**a**) vertical and (**b**) oblique forces.

Regarding the Von Mises stresses on the prosthesis framework, the highest stress with both vertical and oblique loading was that in M2 (Table 6 and Figure 8).

## 4. Discussion

The placement of fixed restorations in patients with atrophic jaws using dental implants is one of the most challenging issues in modern dentistry. Sinus pneumatisation in atrophic maxillae and the distance between the nasal base and alveolar crest can make treatment of these jaws very difficult, which gave rise to the all-on-four treatment concept. In a nutshell, in the all-on-four concept, the two anterior implants are placed axially, while the two posterior implants are placed at an angled position to maximize implant length. This technique avoids anatomic structures and allows the use of longer implants without the need for bone grafting.

Peri-implant bone behavior lacks reference values in the conditions analyzed here. Mechanical stimulus is known to trigger bone remodeling, but a consensus on the exact mechanisms that regulate this process is lacking. According to the mechanostat theory, disuse may result in bone atrophy, while an above-physiological stimulus may lead to bone resorption when deformation exceeds the tolerable limit. Although the exact stimulus for bone response is unknown, overload posed a risk of bone loss around the implants. Moreira de Melo et al. stated that there may be a lower risk of peri-implant bone loss with the use of an implant with a larger (3.5 mm) rather than a narrower (2.9 mm) diameter [11]. The use of asymmetrically placed implants could be preferable to symmetrical but narrower implants.

Finite element analyses have examined standard implant placements when using the all-on-four concept, i.e., two implants placed vertically and anteriorly in the lateral incisor region anteriorly, and two implants placed with the implant neck at an angle in the second premolar region posteriorly [4,7,8,10,17]. However, in practice, two sides of maxillary bone and sinus pneumatization are hardly ever symmetrical. Perfect bilateral symmetry is rarely found, and asymmetries can be attributed to biological and environmental factors like early tooth loss on one side, periapical cyst, or a traumatic extraction. These factors force clinicians to differ from standard concepts. Asymmetrical implant dimensions and angulations could be used. In severe cases, some locations must be passed completely and an asymmetrical positioning of the implants could be necessary.

Anterior implant position can be changed to avoid the need for bone volume augmentation. Likewise, with asymmetric sinus pneumatisation, the implants can be placed as posterior as possible to reduce the cantilever length, as recommended in some studies [2,5,9,18]. We created Models 2 and 3 to evaluate by finite element analysis the stress changes that occur in such asymmetric cases.

Taruna et al. reported that a minimum bone thickness of 5 mm and minimum bone height of 10 mm are required for anterior implants. Posterior implants at angles of 17–45 degrees require a minimum thickness of 4 mm and minimum length of 11.5 mm [19]. Few reports have examined the effects of angulation or the number of implants on stress in all-on-four treatments [1,4,10,14,16,18].

Ozan et al. reported in their study (in which half of a mandible model and 100 N was applied to the molar region vertically) that decreasing the cantilever length by tilting the posterior implants resulted in a reduction in stress values in the peri-implant bone, abutment, prosthetic screw, and metal framework. The groups with 30- and 45-degree tilted posterior implants and shorter cantilever lengths showed better stress distributions in comparison to the groups with straight and 17-degree tilted posterior implants with longer cantilever [10].

Liu et al. (full maxilla model and 150N in the multivectoral direction was applied unilaterally to the cantilever region) also reported better stress distributions were observed in the 30-degree and 45-degree tilted posterior implants with shorter cantilever in comparison to the groups with straight and 17-degree tilted posterior implants with longer cantilever [7].

Carvalho et al. compered two models, in the M1 model two axially inserted anterior implants and two tilted implants, 15 mm in length, placed tangential to the maxillary sinus’s anterior wall were used. In the M2 model, two axially inserted anterior implants and two trans-sinus tilted implants, 24 mm in length, were used. In both models posterior tilted implants were placed at an angle of 30 degrees. For the finite element analysis (FEA), an axial load of 100 N was applied on the prosthesis. The results were similar when the stresses on peri-implant bone were compared: 0.139 and 0.149 for Models 1 and 2. The tension values were lower in the model with trans-sinus implants (M2). Their results suggested that with trans-sinus implants, the emergence of these implants in a posterior position eliminates the extension in the cantilever of the prosthesis and, therefore, increases the polygon of its support, indicating a better mechanical performance of this model [9]. In our study, similar occlusal load (100 N vertical and oblique) and implant tilt (30-degree) were used. Our results support the notion that more posterior placement of distal implants lowers peri-implant bone stresses. In our study, M2 showed lower stress values in cortical bone around implants when compared with other models.

Occlusal contacts form in the area where the food is first taken when chewing starts, while there is no occlusal contact on the contralateral side. Accordingly, in our study, the forces were applied unilaterally [6,7,14].

Although the amplitude of applied load varies among studies, we adopted a 100 N force in agreement with previous research in the literature [4,6,10,15,16]. Equal loads of 100 N were applied to the same region in each model. The magnitude of the load was not important since the models were considered to be linearly elastic. The stress values were acquired to compare the models and not to report the absolute values. Ferreira et al. stated that owing to the linear nature of finite element analysis, the increase/decrease of stress levels is proportional to the applied load [15].

With vertical loading, the highest Pmax was around the implant in the right anterior region in M1, while the value in the same region was very similar in M3. This may be attributed to the lever-arm effect. Biomechanically, regions where the tensile stress is highest are those far from the abutment. Therefore, the highest value for a unidirectional force applied from the posterior region is in the anterior implant. With vertical loading, the lowest Pmin was in the right posterior implant region, in accordance with biomechanical principles.

In this study, we tried to cover the force vector directions that may occur during chewing by applying oblique forces from the same region in the models [14,16,20].

When oblique forces were applied, the highest Pmax of the cortical bone occurred around the right posterior implant in M1; 8.992 Mpa, with a similar force acting around the implant in the same region in M3; 8.780 Mpa. The result in same area in the M2 model has 3.692 Mpa, less than half of M1 (%41).

Examining the Pmin values, the lowest value was that in the implant in the right posterior region of M3. These results obtained with oblique loading were attributed to the force reaching the implants via different vectors.

In the case of full-arch implant-supported fixed dentures, multiple implants are splinted through a metallic infrastructure; thus, the loading in one point of the prosthesis promotes a stress concentration in all implants and surrounding bone in different degrees, which can cause a significant incidence of failures in various parts of the prosthesis, such as screws, abutments, frameworks, and implant.

Some studies have investigated the influence of material properties on the stress distribution in implant-supported fixed prostheses with different occlusal surface materials and infrastructures.

Tribst et al. stated that there is a directly proportional relationship between the fixed full-arch implant-supported prosthesis weight and the strain generated around the osseointegrated implants. Additionally, the CoCr framework with ceramic veneer (≅60 g) is the heaviest among compared materials (Zirconia framework with ceramic veneer ≅40 g, CoCr framework with acrylic resin tooth ≅20 g, titanium framework with acrylic resin tooth ≅15 g, PEEK framework with acrylic resin tooth ≅10 g) [21].

Çalışkan et al. found that metal and Zr showed strain patterns lower than Polyether ether ketone (PEEK) with the all-on-four concept. They reported that increased elastic modulus of the framework reduced the stresses transmitted to the implants and bone [22]. Tribst et al. reported that similar mechanical behavior for the implants and bone was observed with similar materials and model design [23].

Lee et al. performed stress analysis on titanium, Zr and PEEK infrastructures on implants placed according to the all-on-four technique. They reported the highest values in tensile and compression stresses in the bone around the implant in the PEEK substructure. Von Mises stresses within the substructures were less common in PEEK material [24].

In this study results show an increased stress on prostatic parts in M2 and M3. Moving implants more posterior and increasing distance between implants puts extra pressure on both framework and implants themselves while the stress on the bone decreased significantly. Regarding the Von Mises stresses on the prosthesis framework, the highest stress with both vertical and oblique loading was that in M2 (449.54 Mpa vertical and 621.43 Mpa oblique) between the right premolar and incisor implants. The same area in M1 was 199.40 Mpa vertical and 309.43 Mpa oblique, less than half of the results in the M2 model. The results in M3 were in-between (255.52 Mpa vertical and 409.16 Mpa oblique).

In this study, 100 N forces were applied vertically and obliquely (at 45 degrees) over the palatal tubercles of the right first molar unilaterally. The force applied and cantilever length stayed the same for all models. When left posterior implant was placed farther posterior, cortical bone stress on either side decreased. These results suggest that moving implants further back on the arc not only shorten cantilever but also increase balance and help to eliminate some of the tipping forces on opposite sides.

## 5. Conclusions

We conclude that moving the implants posteriorly as far as possible may reduce stress on the bone when applying maxillary all-on-four treatment. In the other scenarios, placing an anterior implant more posteriorly reduced stress on the bone somewhat, but did not produce a major change. We recommend that posterior implants should be placed as posteriorly as possible, although these findings should be validated by clinical studies.

## Figures and Tables

**Table 1:** Elastic modulus values and Poisson ratios of the data input materials.

Materials	Young’s Modulus (MPa)	Poisson Ratio
Cortical bone	13,700	0.30
Spongious bone	1370	0.30
Titanium	110,000	0.35
Cr–Co infrastructure (bar)	218,000	0.33
Acrylic	2700	0.35

**Table 2:** Cortical Bone max Von Misses Stress (Mpa) values under vertical and oblique forces.

Models	Right Posterior	Right Anterior	Left Anterior	Left Posterior
M1 vertical M1 oblique	2.293	7.546	1.387	0.385
	8.992	8.246	0.508	0.635
M2 vertical M2 oblique	1.626	2.652	0.626	0.143
	3.692	3.641	0.913	0.314
M3 vertical M3 oblique	2.159	7.349	1.195	0.219
	8.780	7.384	0.824	0.359

**Table 3:** Implant neck region max Von Misses Stress (Mpa) values under vertical and oblique forces.

Models	Right Posterior	Right Anterior	Left Anterior	Left Posterior
M1 vertical M1 oblique	79.37	82.34	16.41	8.61
	107.89	81.39	20.32	9.03
M2 vertical M2 oblique	125.13	123.41	53.70	4.76
	154.41	269.60	68.87	35.61
M3 vertical M3 oblique	79.83	76.83	6.87	4.62
	107.42	88.59	10.74	6.90

**Table 4:** Abutment neck region max Von Misses Stress (Mpa) values under vertical and oblique forces.

Models	Right Posterior	Right Anterior	Left Anterior	Left Posterior
M1 vertical M1 oblique	162.15	41.14	11.87	32.73
	253.98	80.93	22.02	20.08
M2 vertical M2 oblique	175.98	76.73	45.47	12.22
	238.69	116.53	25.95	23.19
M3 vertical M3 oblique	251.88	25.22	8.88	8.76
	247.50	48.93	12.73	9.62

**Table 5:** Abutment screws max Von Misses Stress (Mpa) values under vertical and oblique forces.

Models	Right Posterior	Right Anterior	Left Anterior	Left Posterior
M1 vertical M1 oblique	30.00	27.77	16.29	16.02
	65.26	37.63	13.96	15.62
M2 vertical M2 oblique	40.39	19.70	4.38	5.55
	36.38	17.89	6.24	11.13
M3 vertical M3 oblique	31.23	26.38	5.72	13.65
	65.32	35.09	3.47	10.93

**Table 6:** Framework Max Von Misses Stress (Mpa) values under vertical and oblique forces.

Framework Max Von Misses Stress (Mpa)	Model 1	Model 2	Model 3
P_max_ vertical P_max_ oblique	199.40	449.54	255.52
	309.43	621.43	409.16
